# Polycystic Ovary Syndrome as Metabolic Disease: New Insights on Insulin Resistance

**DOI:** 10.17925/EE.2023.19.1.71

**Published:** 2023-05-17

**Authors:** Alessandro D Genazzani, Andrea R Genazzani

**Affiliations:** 1. Gynaecological Endocrinology Center, Department of Obstetrics and Gynaecology, University of Modena and Reggio Emilia, Modena, Italy; 2. Department of Obstetrics and Gynaecology, University of Pisa, Pisa, Italy

**Keywords:** Familial diabetes, hyperinsulinemia, inositols, insulin resistance, lipoic acid, non-alcoholic fatty liver disease, polycystic ovary syndrome

## Abstract

Polycystic ovary syndrome (PCOS) is a very frequent disease that affects reproductive ability and menstrual regularity. Other than the criteria established at the Rotterdam consensus, in these last few years a new issue, insulin resistance, has been found frequently, and at a very high grade, in patients with PCOS. Insulin resistance occurs for several factors, such as overweight and obesity, but it is now clear that it occurs in patients with PCOS with normal weight, thus supporting the hypothesis that insulin resistance is independent of body weight. Evidence shows that a complex pathophysiological situation occurs that impairs post-receptor insulin signalling, especially in patients with PCOS and familial diabetes. In addition, patients with PCOS have a high incidence of non-alcoholic fatty liver disease related to the hyperinsulinaemia. This narrative review focuses on the recent new insights about insulin resistance in patients with PCOS, to better understand the metabolic impairment accounting for most of the clinical signs/symptoms of PCOS.

Polycystic ovary syndrome (PCOS) is a very common disease, with an incidence of 5-21% in women during their fertile life (18–45 years of age) worldwide.^1^ PCOS is clinically diagnosed when two of the three 2003 Rotterdam consensus criteria are met: (i) chronic anovulation disorder (in the form of oligo-or anovulation up to amenorrhoea); (ii) clinical (e.g. acne, hirsutism) or biochemical signs of hyperandrogenism; and (iii) micro-polycystic ovaries, i.e. ≥20 follicles per ovary and/or an ovarian volume ≥10 mL on either ovary (detected using ultrasound transducers with a frequency bandwidth that includes 8 MHz).^2–4^ Combining these three criteria highlights four distinct phenotypes of PCOS, the most clinically severe of which is the one that is positive for all three criteria.^5^

Although the overall prevalence of PCOS is similar across all countries, a significant variability due to ethnic factors has been found to influence the phenotypic manifestations of the syndrome; for example, the prevalence of PCOS among Caucasian women ranges from 4.7 to 6.8%.^6^ Furthermore, it has been reported that, when groups move from one place to another, they retain their ethnic predisposition to PCOS and to impaired metabolism sustained by hyperinsulinaemia and/or diabetes, as observed, for example, in British women of Indian subcontinent Asian origin, who present a higher prevalence of PCOS and type 2 diabetes.^6^ From these data, we may infer that there is an environmental as well as genetic component to PCOS, because diet, exercise, and lifestyle have wide ethnic variations.

Insulin resistance (IR) is a specific biological adaptation that induces a compensatory hyperinsulinaemia in approximately 70-80% of women with PCOS and central obesity, and in 15-30% of lean women diagnosed with PCOS.^3,7,8^ IR is frequently found in patients with PCOS, regardless of their body mass index, considering that up to 50% of patients with PCOS are overweight or obese. Notably, overweight and obesity are frequently observed in patients with PCOS phenotype A, the most severe of the four phenotypes.^5^

Consequently, these metabolic features (i.e. insulin plasma level evaluation, body weight or body mass index computation) should be considered when evaluating patients with PCOS. As such, two alternative types of PCOS have been suggested: the classic reproductive phenotype of PCOS and a new one, with high metabolic risk and impairment, whose proposed name is ‘metabolic reproductive syndrome’.^9^ Due to the relevance that the metabolic aspects may play in PCOS, this review will focus on the new issues recently highlighted.

## Insulin actions and insulin resistance

**Figure 1:**
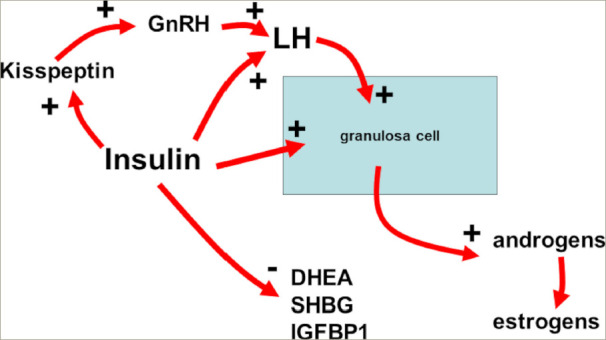
Schematic summary of insulin actions on the reproductive axis

The role of insulin in mammals is extremely important. In humans, insulin is the main regulator of glucose homeostasis, inducing the uptake of glucose in all tissues, in particular in the adipose tissue, muscles, heart and liver. Insulin also decreases lipolysis, thereby reducing the amount of free fatty acid in the blood, which partly modulates the effects of insulin on glucose production by the liver.^10^ Other than these metabolic effects, insulin plays a role as a co-gonadotropin. Specifically, it modulates the ovarian function by amplifying the action of the luteinizing hormone (LH) on theca cells, thus participating in the androgen secretion of androstenedione from the ovaries.^11^ Such co-gonadotropic effects are partly direct and partly indirect since insulin facilitates the expression of its own receptors on the granulosa cells but also on the LH and insulin-like growth factor 1 receptors. When IR occurs, the increased plasma levels of insulin result in excessive stimulation of the ovaries, thus inducing an overproduction of androgens.^10,11^ It is clear that excess insulin impairs not only ovarian function but also the central and neuroendocrine control of the reproductive axis (*Figure 1*).

*Insulin plays specific roles centrally on gonadotropin-releasing hormone through a positive action on kisspeptin release thus inducing luteinizing hormone secretion. At the ovarian level, insulin amplifies luteinizing hormone signalling and improves androgen production. Insulin also plays a role in sexual hormone-binding globulin production and in dehydroepiandrosterone release from the adrenal gland.*

*DHEA = dehydroepiandrosterone; GnRH = gonadotropin-releasing hormone; IGFBP1 = insulin-like growth factor binding protein 1 ; LH = luteinizing hormone; SHBG = sex hormone-binding globulin.*

**Figure 2:**
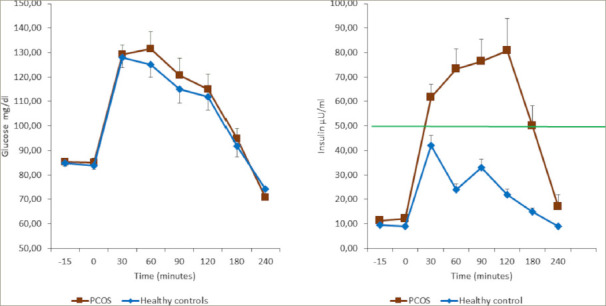
Glucose (panel A) and insulin (panel B) response to the oral glucose tolerance test in a group of patients who are obese with polycystic ovary syndrome (n=25) and healthy controls who are obese (n=23)

Indeed, recent studies have reported the link between kisspeptin and the gonadotropin-releasing hormone (GnRH)-i nduced secretion of LH, as evidenced by the concomitant secretion of the pulsatile peaks of both hormones.^12,13^ Moreover, kisspeptin and LH have been found to be cosecreted in eumenorrheic PCOS but not in oligomenorrheic PCOS.^14^ In addition, insulin plasma levels were found to significantly correlate with both kisspeptin and LH plasma levels in subjects with PCOS and patients with functional hypothalamic amenorrhoea (FHA).^13,15^ All these data support the hypothesis that insulin plays a relevant role in the control of reproduction in healthy conditions as well as in physiopathological states such as FHA^16^ and PCOS in patients who are overweight/obese.^15–17^ Any excess of insulin can interfere with GnRH secretion at the hypothalamic level through an excess of kisspeptin, thus causing excessive stimulation of the gonadotropin release and LH oversecretion.^14^ The opposite is true in the case of FHA.^15^ In fact, patients with FHA show very low levels of insulin that correlate with very low levels of LH.Consequently, insulin plasma levels must not be elevated in the blood, and any excess might induce specific effects not only on the metabolic but also on the reproductive side. Insulin’s role is to keep glucose concentrations under control; such a task, however, is accomplished by a highly sophisticated network of hormones and neuropeptides released mainly from the pancreas (i.e. insulin) but also from the brain, liver and intestine, as well as from the adipose and the muscle tissue. Within this network, the pancreas plays a key role by secreting the blood sugar-l owering hormone insulin and its opponent glucagon.^18^ When such balance between insulin and glucagon is not in equilibrium and plasma insulinemia rises in the presence of normal glucose plasma levels, IR takes place; this means that higher insulin levels are required to maintain a normal level of glucose into the blood flow, to maintain the glucose homeostasis (*Figure 2*).^18,19^

IR occurs whenever the ability of insulin to induce its specific metabolic actions is reduced and the metabolic uptake and production of glucose and lipolysis is impaired. Consequently, a compensatory mechanism of releasing higher levels of insulin takes place, and higher levels of insulin are released both in the baseline and after glucose load.^8,10^

IR has specific negative effects on some organs and tissues. In the liver and skeletal muscle, IR increases lipolysis by the accumulation of non-esterified fatty acids. When lipids are accumulated inside the hepatocytes, they activate the diacylglycerol/protein kinase C and inhibit the insulin receptor.^10^ Inside the skeletal muscles, IR probably induces the inhibition of both the phosphoinositide-3 kinase and the phosphorylation of insulin receptor substrate 1, thus changing the expression of the glucose transporter-4 (GLUT-4) vesicles and reducing glucose upload.^20,21^

Notably, IR occurs more frequently in patients with PCOS who are overweight/obese, especially in those with central obesity;^4^ however, an extensive literature search demonstrated that IR is also possible in normal-weight PCOS, regardless of body mass index.^8,22^ The evidence that IR occurs in lean patients with PCOS suggests the hypothesis that a post-receptor defect could be affecting glucose upload,^8,23,24^ rather than an excessive serine phosphorylation of the insulin receptor.^8,25^ Being overweight or obese is central for the onset of several of the classic PCOS symptoms, as was recently shown by the meta-analysis by Behboudi-Gandevani et al.^26^ Notably, abnormal glucose control and/ or type 2 diabetes have also been observed to develop more rapidly in patients with PCOS than in the control patients.^27^ This evidence reinforces the suggestion that patients with PCOS should undergo the oral glucose tolerance test (OGTT),^28^ a method for measuring the body's response to glucose that lasts at least 2 hours, to disclose the insulin response under metabolic stress. OGTT discloses the patient's hyperinsulinaemic condition when insulin response is higher than 50 µU/ml within 30–90 minutes from the glucose load.^19,29^

*Though the glucose response was similar in both groups, the insulin response was higher in subjects with polycystic ovary syndrome. This observation suggests that, in patients who are obese with polycystic ovary syndrome, higher levels of insulin are needed to maintain normal glycaemic control. It cannot be excluded that such hyperinsulinaemic response might be due to a reduced insulin clearance . The green line represents the actual maximal insulin response under oral glucose tolerance test considered within normality (figure drawn using data from Genazzani et al.).*^19^ *PCOS = polycystic ovary syndrome.*

### Insulin signalling and insulin resistance: role of inositols

It is a common observation that a high percentage of patients with PCOS undergoing treatment with insulin sensitizers (i.e. metformin) experience significant improvement not only in the metabolic and hormonal parameters, mainly the hyperinsulinaemic state, but also in hyperandrogenic signs, with the recovery of an almost normal ovarian and menstrual function.^17,30,31^ Unfortunately, metformin dosage depends on both the grade of obesity of the patient and on their hyperinsulinaemic condition; moreover, the higher the dosages are, the more frequent the gastrointestinal side effects.^17,30,31^ A defect in the inositol phosphoglycan (IPG) second messenger pathway could explain the appearance of hyperinsulinaemia via impairment of the post-receptor insulin-i nduced signal; this observation is the basis for the proposal of new therapeutical strategies to manage hyperinsulinaemia in patients with PCOS.^32,33^ Indeed, IPGs represent an essential biological step in the transmission of the specific hormonal and metabolic signals generated after insulin links to its membrane receptor. IPGs are produced at the cellular membrane level after the hydrolysis of glycosyl-phosphatidylinositol lipids located on the internal surface of the cell membrane;^34^ then, IPGs are part of the post-receptor intracellular mechanism that corresponds to the second messenger. This insulin-i nduced intracellular mechanism controls the oxidative and non-oxidative metabolism of glucose and the uptake of glucose by GLUT-4 from the extracellular environment.^9,35^ For this reason, in recent years, inositols have been used consistently as an integrative strategy for improving the cellular response to the metabolic cascades that are activated by insulin binding to its receptor. However, insulin is not the only hormone to use IPG; other peptide hormones, such as thyroid-stimulating and follicle-stimulating hormones, use them as second messengers.^34,36^

Inositols belong to a family of nine isomers, only two of which are relevant for the mechanisms described above: myo-i nositol (MYO) and D-chiro inositol (DCI). Inositols are found in many plants, vegetables such as beans, and fruits. Though belonging to the vitamin group, inositols have a chemical formula similar to glucose and can be produced by our human biology, but most of the inositol comes from food.^8,37^ Once inositol enters the cells, it is transformed into phosphatidil-myo-i nositol and then into inositol-triphosphate, which is the real second messenger of peptide hormones (i.e. insulin, thyroid-stimulating hormones and follicle-stimulating hormones).^34,38^ According to Larner et al.,^39,40^ a specific balance is needed between two out of the nine inositol isomers, that is, between MYO and DCI. These inositol isomers differ only in the position of one hydroxyl group, and DCI derives from MYO thanks to the activity of epimerase.^40^

As mentioned, a specific balance is needed between MYO and DCI.^39,40^ Specifically, both MYO and DCI are relevant to controlling the correct transmission of the metabolic signal of insulin after it binds to its membrane receptor. Once insulin binds to a receptor, it activates a specific kinase cascade, which in turn activates the phosphorylation of the protein kinase B/Akt; this determines the translocation of GLUT-4 vesicles on the cell membrane to upload glucose.^40^ In addition, insulin binding determines another cascade of events that converts MYO to DCI through the activation of the phospholipase and epimerase enzymes.^35^ DCI induces glycogen synthase in the cytoplasm to transform glucose into glycogen; at the same time, it activates the mitochondrial pyruvate dehydrogenase phosphatase, which induces the oxidation of glucose inside the mitochondria.^8,35^ It is known that the role of the inositols, MYO and DCI, is relevant from a biological point of view. Maintaining a perfect equilibrium between the two allows for an adequate upload of sugar from the outside of the cell; through the oxidation of glucose inside the mitochondria and the transformation of glucose into glycogen, a gradient of concentration that allows for an additional upload of glucose from outside the cell through the GLUT-4 vesicles is achieved.^8^

As can be imagined, whatever changes the equilibrium between MYO and DCI impacts the upload of glucose and its transformation into glycogen or oxidation inside the mitochondria. In particular, events that can negatively affect epimerases reduce DCI production and impair glucose upload, slowing glucose upload and then increasing insulin (i.e. hyperinsulinaemia) to compensate for the impaired MYO-to-DCI conversion.

Indeed, studies conducted on both animals and humans with diabetes reported that higher amounts of MYO are present in urinary excretion, while DCI is reduced.^33,41^ This finding is suspected to be explained by a reduced expression and/or bioactivity of the epimerase.^42^ Consequently, these data support the hypothesis that IR occurs due to an abnormal enzymatic expression, rather than due to other putative reasons.^8^ As a demonstration of abnormal enzymatic expression, various studies have focused on the role played by inositols on insulin sensitivity.^43^ Although MYO effectively reduces IR under OGTT in normal-weight PCOS,^22^ a differential IR was observed in patients with PCOS who are overweight or obese.^19^ This finding raises the possibility that specific factors (i.e. the impaired epimerase expression) might be at the basis of the differential activity of the epimerase enzyme. Considering previous reports about altered DCI production and urinary excretion,^33,41^ a clear biological scenario was observed when DCI was administered to patients with PCOS who are overweight or obese.^44^ All patients demonstrated reduced insulinaemia under the integrative treatment and improved IR, as shown with the OGTT; however, the subjects that reported the presence of familial diabetes in first-degree relatives (parents and/or grandparents) showed a higher IR with the OGTT and greater improvement under treatment.^44^ These data support the hypothesis that DCI integration positively corrects a reduced endogenous DCI production in patients with familial diabetes.

Considering that diabetes might represent a serious predisposing factor in reducing insulin sensitivity and increasing IR and hyperinsulinemia, it has been suggested that the administration of inositols may depend on the ability of epimerase to convert MYO into DCI in an adequate quantity.^45,46^ In the absence of such familial predisposition, the administration of MYO is plausible alone^47^ or in combination with DCI;^48^ nonetheless, DCI integration plays a relevant role in the presence of familial diabetes.^8,43–46^

### Alpha-lipoic acid: the silent element that drives insulin sensitivity

Considering the complexity of human biology, it is not possible that only inositols are responsible for controlling the cellular uptake of glucose and, thus, for controlling glycaemia in the biological fluids. Indeed, another compound has very recently been considered relevant for treating IR: alpha-l ipoic acid (ALA). In animal models, ALA has been found to modulate and increase glucose use by increasing adenosine monophosphate-activated protein kinase in skeletal muscles, thus increasing GLUT-4.^49–51^

ALA, also known as thioctic acid, is a naturally occurring substance that is essential for the function of several enzymes in oxidative metabolism.^52,53^ The first clinical use of ALA was described in 1959 for the treatment of mushroom, especially *Amanita phalloides*, poisoning.^54^ ALA is commonly found in vegetables such as spinach and broccoli, and in tomato and meats. In mammals, ALA is synthesized by mitochondria lipoic acid synthase (LASY), which can be downregulated in different clinical conditions, such as diabetes.^55^ ALA and/or its reduced form, dihydrolipoic acid, have many biochemical functions; they act as biological antioxidants (such as metal chelators), which reduce the oxidized forms of other antioxidant agents (such as vitamin C and E and glutathion), and module the signalling transduction of several pathways (such as insulin and nuclear factor kappa B).^51^

Among many actions, ALA improves endothelial dysfunction^56^ and reduces oxidative stress following exercise training,^57^ protecting against the development of atherosclerosis.^57^ Due to these actions, ALA could potentially be considered a therapeutic agent for many chronic diseases with a great epidemiological, economic and social impact, such as hypertension,^58^ Alzheimer’s disease,^59^ cognitive dysfunction, and diabetes mellitus and its complications.^55,60^

Consequently, ALA has been proposed as a therapeutic compound for many diseases, especially diabetes and PCOS, since both present an abnormal dysmetabolic profile.^61,62^

Recently, several studies showed the great efficacy of ALA in controlling insulin sensitivity. Indeed, we recently found that ALA administration at a dose as low as 400 mg every day was effective in reducing IR and improving insulin sensitivity in patients with PCOS, as demonstrated by the significant decrease in the homeostasis model assessment index, especially in patients with PCOS with familial diabetes.^63^ These data support the suggestion that ALA administration overcomes the impairment typically present in familial diabetes that downregulates the expression of LASY inside the mitochondria of mammals and in humans.^64,65^ Indeed, reduced endogenous ALA synthesis induces a lower glucose uptake in skeletal muscle cells, which are at the basis of IR.^65^ This defective action decreases adenosine monophosphate-activated protein kinase in skeletal muscles,^45^ thus reducing induction on GLUT-4.^49,65^ However, this is not the only benefit. Notably, only patients with familial diabetes showed transaminase plasma levels close to the upper levels of normality and higher than patients with no familial diabetes.^66–68^ ALA administration to patients with familial diabetes significantly decreased transaminase to normal plasma levels. These data consistently support the hypothesis that the integrative administration of ALA (mimicking endogenous ALA) eliminates most of the metabolic impairment in subjects with PCOS, especially in those with familial diabetes.

The use of ALA as treatment has great clinical significance. A recent review states that non-alcoholic fatty liver disease (NAFLD) is found in a high percentage (from 40 to 70%) of patients with PCOS,^69^ and the combination of PCOS with obesity and IR is dangerous as it triggers not only NAFLD but also, with a great probability, the occurrence of type 2 diabetes.^69,70^ The ability of the ALA administration to reduce transaminase levels in patients with familial diabetes confirms its ability in liver protection, as previously reported,^54^ and suggests that ALA positively affects liver function in patients with PCOS, by reducing the risk of developing a liver impairment such as NAFLD and, later, type 2 diabetes.^63^ This contradicts another report that did not sustain such effects.^71^

For these reasons, and considering the benefits of inositols (i.e. MYO and DCI), ALA has been coupled with MYO or DCI to improve the beneficial effects on insulin sensitivity. Various studies have shown that the combination of ALA with MYO or DCI significantly improved both reproductive and metabolic outcomes in patients with PCOS with and without familial diabetes.^47,66,67^ Indeed, because patients with PCOS with familial diabetes present reduced expression of LASY and of epimerase, the recovery of insulin sensitivity and reproductive function seems to be greatly improved by the combination of ALA with DCI,^67^ which is greatly effective also in the absence of familial diabetes.^67^ The combination of ALA and MYO also improves the reproductive axis and metabolic impairment; however, in the case of familial diabetes, it does not overcome the reduced expression/function of epimerase.^47,66^

**Figure 3:**
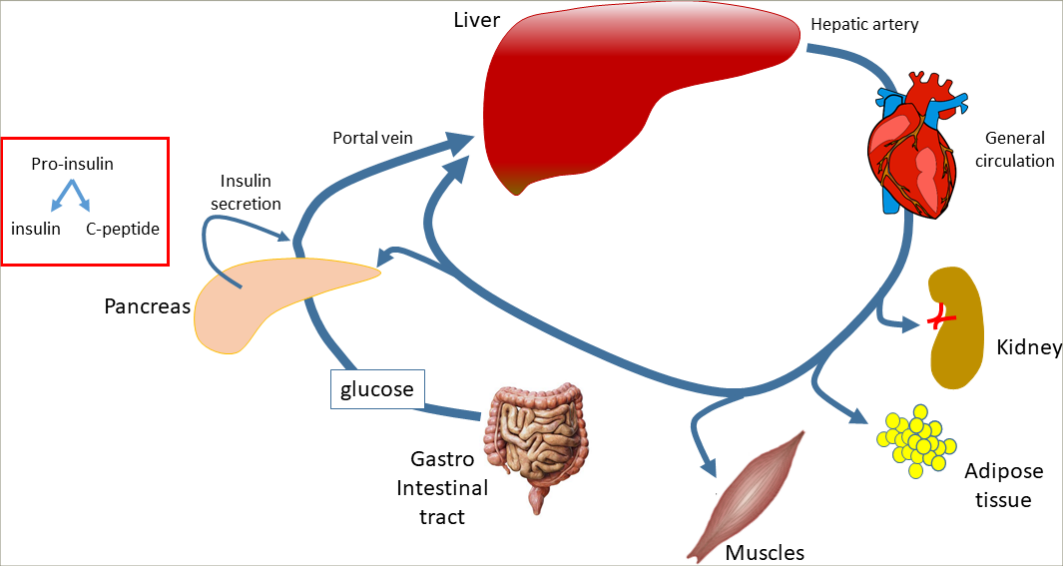
Insulin secretion and removal from the general circulation depend on pancreatic activity and from the clearance exerted by various organs: liver (-50%), kidney (-30%), muscles (-15 to -35%)

*Insulin secretion from* β *cells of Langherhans islets of the pancreas derives from cleavage of pro-insulin into insulin and C-peptide, and the these products are secreted into the portal vein. The blood passes through the liver, which extracts about 50% of the newly secreted insulin. The remaining insulin exits from liver and enters into the systemic arterial circulation via the hepatic veins. The remaining amount of insulin is removed while passing through the kidneys and the muscles. Whenever liver clearance ability is impaired, a lower amount of insulin is degraded.*

### Hepatic insulin extraction as an index of hepatic involvement in insulin resistance in polycystic ovary syndrome

A classic silent and asymptomatic hepatic disease is NAFLD. NAFLD is frequently but not always characterized by elevated aminotransferases (i.e. alanine aminotransferase [ALT] and aspartate aminotransferase [AST]),^69^ which are considered relative indicators of the presence of NAFLD, since they are eventually close to the upper range of normality.^46,72^ There is growing evidence that NAFLD and PCOS share the same metabolic triggering factors,^70^ and that NAFLD is related more to IR than to liver fat content.^73,74^ The incidence of NAFLD in fertile women with PCOS has been shown to be correlated with IR, altered lipid profiles and androgen plasma levels.^75–78^ Although androgen levels might not differ between patients with PCOS and female patients with NAFLD and without PCOS,^79–81^ lower levels of sexual hormone-binding globulin have been proposed as a putative mediator between IR and NAFLD; sexual hormone-binding globulin is a steroid transporter and an indicator of the metabolic and nutritional status, whose hepatic production is regulated by insulin.^69,82^ Therefore, it is clear that there is a close relation between IR and hyperinsulinaemia and the possible occurrence of a hepatic functional impairment, such as NAFLD, which is probably associated with increased transaminase plasma levels.

Consequently, several reports have demonstrated that insulin clearance is impaired in patients who are overweight or obese,^83^ similarly to patients with PCOS who are overweight or obese.^84,85^ Recently, our group evaluated the hepatic insulin extraction (HIE) index in patients with PCOS who are overweight or obese and found that this index indicated impairment, specifically when familial diabetes was present in at least one first-degree relative (parents and/or grandparents).^84,85^

HIE can be computed with various algorithms,^73,86^ of which the computation of the ratio between insulin and C-peptide plasma levels is the simplest.^86^ Recent studies reported that reduced liver function and the ability to clear insulin participate in improving IR.^73,86,87^ From a physiological point of view, HIE reflects insulin kinetics intended as the balance between synthesis from the pancreas and clearance exerted by the liver. Since C-peptide clearance by the liver is almost negligible, C-peptide can be intended to reflect the pancreatic synthesis of insulin.^88^ Indeed, at the pancreatic level, pro-i nsulin cleavage generates one insulin molecule and one C-peptide molecule. Although at the pancreatic level the production ratio between insulin and C-peptide is 1, this ratio can be computed using the circulating levels of both peptides. This way, the ratio greatly reflects the balance between insulin hepatic clearance kinetics and insulin pancreatic release, which corresponds to C-peptide concentrations, since its hepatic clearance is very low.^89^ The liver function, in general, extracts up to 50% of the insulin delivered by the pancreas, while the kidneys extract 30% and the muscles extract approximately 15–35% of what remains in the circulation after the first pass through the liver (*Figure 3*).^83,90–93^ The HIE depends on the adequate expression/synthesis of the insulindegrading enzyme.^88,94^

HIE has been evaluated in patients with PCOS who are overweight or obese, while accounting for whether there was anyone with diabetes among first-degree relatives (i.e. parents and/or grandparents).^85^

One study found not only that all subjects with PCOS with familial diabetes had higher insulin plasma levels in baseline conditions after overnight fasting, but that they also showed higher HIE than subjects with PCOS without familial diabetes.^84^ In addition, patients with PCOS with familial diabetes also showed ALT and AST plasma levels at the upper limit of normality and higher than in patients with PCOS without familial diabetes.^85^ These data let us infer that a specific impairment might be induced by a familial predisposition to diabetes affecting hepatocyte functions. Indeed, patients with PCOS with familial diabetes showed a higher IR than patients with PCOS without familial diabetes when undergoing OGTT.^84^ As C-peptide response did not differ between the two PCOS groups, HIE resulted higher in patients with PCOS with familial diabetes for almost the whole duration of the OGTT, mainly due to the reduced hepatic clearance of insulin.^84^ Recently, the integrative approach to treating PCOS using ALA alone^63^ or in combination with MYO^66^ or DCI^67^ demonstrated great efficacy, especially in patients with PCOS with a familial predisposition to diabetes.^68^

These results support the hypothesis that the familial predisposition to diabetes somehow impairs not only the peripheral insulin sensitivity through the lower/defective expression/synthesis of both epimerase and LASY, but also the hepatic ability to clear insulin. In the absence of familial diabetes, only overweight or obesity are responsible for the peripheral defect in insulin sensitivity, thus inducing a compensatory greater insulin (and C-peptide) production; however, when familial diabetes is present, the impaired function/ synthesis of epimerase and LASY is additional to the defect in hepatic clearance, due to a defect of insulin-degrading enzyme function/expression. The combination of these events determines a higher amount of circulating insulin (due to the overproduction and lower clearance) in PCOS with familial diabetes than in PCOS without familial diabetes. The frequent occurrence of elevated (though not pathological) ALT and AST plasma levels has to be attentively taken into consideration, this being a signal of hepatic impairment. The combination of hyperinsulinemia and elevated transaminase has been considered a trigger for NAFLD, which occurs more frequently in patients with PCOS than in the normal population.^69^

In conclusion, this narrative review recognizes the importance of adopting a precise approach to the treatment of PCOS, not only from a gynaecological perspective but also from an internal medicine perspective. The evidence indicates that there is a greater incidence of dysmetabolic diseases, such as diabetes and dyslipidaemia, together with NAFLD and non-alcoholic steatohepatitis in patients with PCOS, especially in those with familial diabetes; therefore, patients with PCOS deserve a careful evaluation. Every time these patients refer to the gynaecologist for any kind of reproductive impairment or just for menstrual irregularity, a metabolic evaluation has to be done together with the hormonal reproductive profile.
